# The complete mitogenome of *Chlorops oryzae* Matsumura (Diptera: Chloropidae)

**DOI:** 10.1080/23802359.2021.1934171

**Published:** 2021-06-03

**Authors:** Jia Wang, Xian-Ya Li, Run-Bang Du, Ying-Hong Liu

**Affiliations:** College of Plant Protection, Southwest University, Chongqing, China

**Keywords:** Mitogenome, *Chlorops oryzae*, phylogenetic analysis

## Abstract

*Chlorops oryzae* Matsumura is an important pest of rice plants throughout Asia, and has even become a major pest in some regions. Here, we present the complete mitogenome of *C. oryzae* for the first time. The complete mitogenome is 17,313 bp in length and contains 37 genes (13 protein-coding genes, 22 transfer RNAs, and two ribosomal RNAs) and a control region. The overall base composition is 42.04% for A, 37.18% for T, 12.59% for C, and 8.29% for G, with a bias toward A + T (79.22%). Protein-coding genes *cox1* features an atypical ACG start codon and *cox2*, *nad5*, and *nad4* have incomplete stop codons T or TA. All tRNA genes present the typical clover leaf secondary structure except *trnS1* (AGN), where the DHU arm is replaced by a loop. Phylogeny showed that *C. oryzae* was placed as the basal lineage in Brachycera clade, and shared a closer relationship to Acalyptrate species.

*Chlorops oryzae* is an important pest of rice plants throughout Asia, and has even become a major pest in some regions. In recent years, the frequent outbreaks of this pest have increasingly aroused concerns in Asia. However, the scientific research on this species is still scarce, and was mainly focused on ecology and physiology (Takeda and Nagata 1992; Takeda 1997, 1998). Till recently, the studies on its genetics was performed (Qiu et al. 2018; Zhou et al. 2020). In this study, the first complete mitogenome of *C. oryzae* was sequenced and provided, which could facilitate further extensive study on this pest, such as evaluation of genetic structure of different geographic populations in an attempt to reveal the genetic mechanisms underlying frequent outbreaks in specific regions (Zhou et al. 2020).

All specimens were collected from Qianjiang county (E 108°43′7″, N 24°9′23″), Chongqing, China, and the voucher specimen was deposited at the Insect Herbarium of College of Plant Protection, Southwest University (Jia Wang, aimarjia@126.com), under the accession number SWU Di-15-72. The total genomic DNA was extracted using SteadyPure Universal Genomic DNA Extraction kit (Accurate Biology, Changsha, China). Twenty-three pairs of primers were designed to amplify the fragments of mitogenome, which were sequenced using Sanger sequencing method and were assembled by DNAMAN 9 (Lynnon Co., San Ramon, CA). The complete mitogenome sequence was annotated by MITOS (Bernt et al. 2013) with manual correction, and was deposited in GenBank under the accession number MW438309.

The complete mitogenome of *C. oryzae* was 17,313 bp in length, and the overall base composition is 42.04% for A, 37.18% for T, 12.59% for C, and 8.29% for G, with a bias toward A + T (79.22%). The structural organization of mitogenome is consistent with the typical model of dipteran insects (Mei et al. 2012; Zhang et al. 2014), including 13 protein-coding genes (PCGs), two ribosomal RNA (rRNA) genes, 22 transfer RNA (tRNA) genes, and a control region (D-loop). Most of genes are located on the H-strand except *trnQ*, *trnC*, *trnY*, *trnF*, *trnH*, *trnP*, *trnL* (TAG), *trnV*, *nad5*, *nad4*, *nad4l*, *nad1*, *rrnL*, and *rrnS*, which are located on the L-strand. All PCGs start with ATN, except for *cox1* with a start codon ACG. Most of PCGs use typical stop codon TAA or TAG, while *cox2*, *nad4*, and *nad5*, are deduced to use incomplete stop codon T or TA, for which the missing A residues required for generating TAA stop codon are provided by post-transcriptional polyadenylation (Markova et al. 2015). The lengths of 22 tRNA range from 63 bp (*trnC*) to 72 bp (*trnV*). All tRNAs display a typical clover-leaf secondary structure except *trnS1* (AGN), where the DHU arm is replaced by a loop. Two rRNA genes, *rrnL*, and *rrnS*, are 1289 bp and 787 bp in length, respectively, and are located between *trnL1* and control region with a separation by *trnV*. The control region is located between *rrnS* and *trnI*, and has a size of 2359 bp.

To illustrate the phylogenetic status of *C. oryzae*, a phylogeny was constructed based on the complete mitogenomes of *C. oryzae* and 24 additional species (Figure 1), using maximum-likelihood (ML) analyses by W-IQ-TREE (Trifinopoulos et al. 2016). Three clades were generated in the phylogenetic tree, representing Brachycera, Nematocera, and outgroup, respectively. In Brachycera clade, *C. oryzae* and three Agromyzidae species were clustered together, and shared a closer relationship to Acalyptrate species, which is consistent with the previous phylogenetic studies of Diptera (Wiegmann et al. 2011).

**Figure 1.**
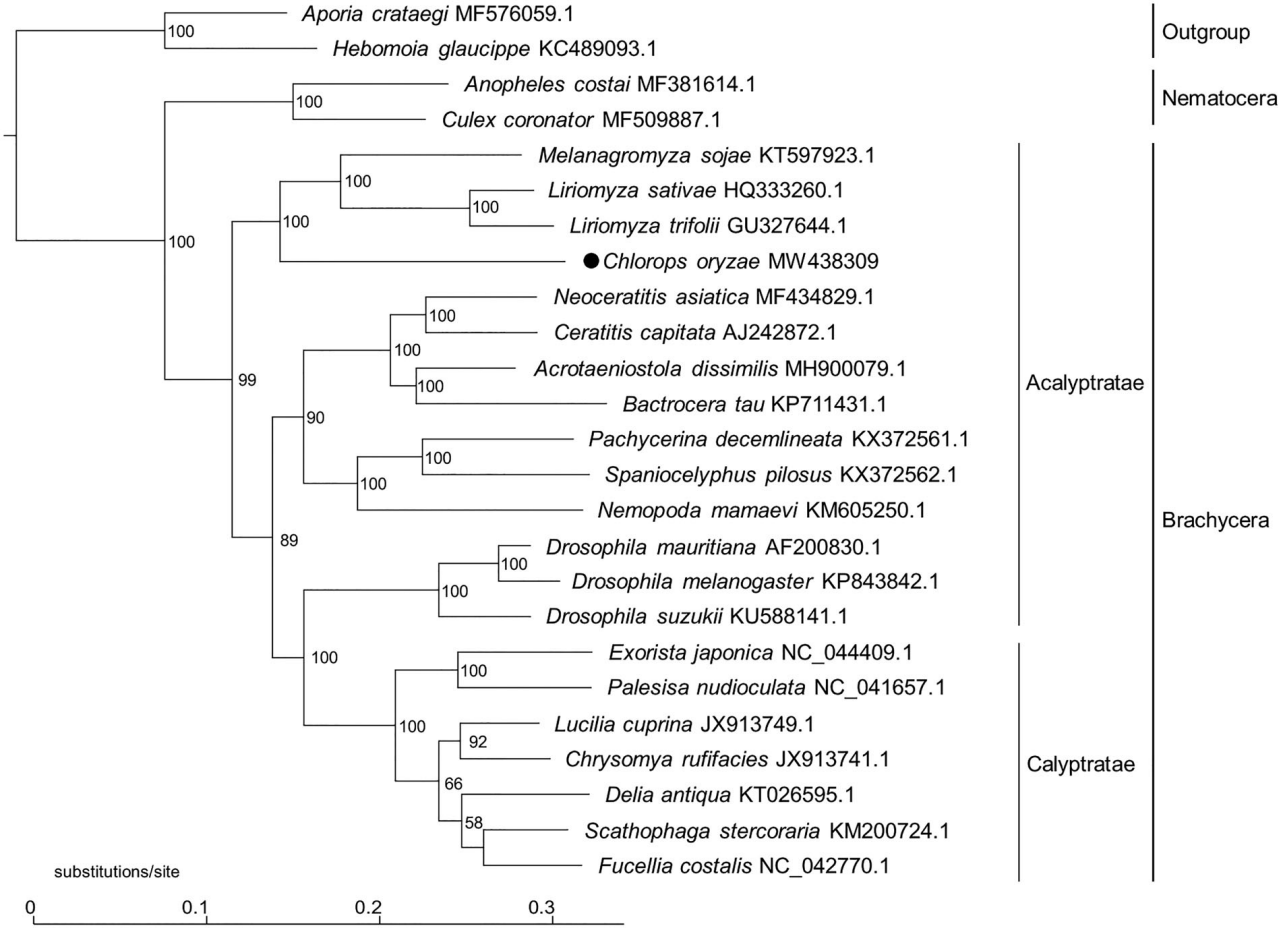
Phylogenetic tree constructed based on complete mitogenomes of *C. oryzae*, 22 additional dipteran species, and two outgroup Lepidopteran species, using maximum-likelihood (ML) methods under general time reversible (GTR) model with 1000 bootstrap replications. Numbers near the branches indicate the bootstrap support values. GenBank accession number for sequences is incorporated. The black dot indicates the *Chlorops oryzae* analyzed in this study.

## Data Availability

The data that support the findings of this study are openly available in GenBank of NCBI at https://www.ncbi.nlm.nih.gov, reference number MW438309.
